# Allocation of National Institutes of Health Funding by Disease Category in 2008 and 2019

**DOI:** 10.1001/jamanetworkopen.2020.34890

**Published:** 2021-01-27

**Authors:** Jeromie M. Ballreich, Cary P. Gross, Neil R. Powe, Gerard F. Anderson

**Affiliations:** 1 Department of Health Policy and Management, Johns Hopkins Bloomberg School of Public Health, Baltimore, Maryland; 2 Department of Internal Medicine, Yale University School of Medicine, New Haven, Connecticut; 3 Priscilla Chan and Mark Zuckerberg San Francisco General Hospital, San Francisco, California

## Abstract

**Importance:**

Prior research suggests an association between burden of disease and National Institutes of Health (NIH) funding. The allocation of NIH funding should reflect, to some extent, the health needs of the population, along with other factors.

**Objective:**

To examine the factors associated with NIH funding in 2019 for 46 diseases.

**Design, Setting, and Participants:**

This cohort study used disability-adjusted life-years to measure the 2008 and 2019 US burden of disease and compared them with NIH categoric funding for 46 diseases.

**Exposures:**

Disability-adjusted life-years to measure the 2008 and 2019 US burden of disease, 2016 health spending, and 2008 NIH funding levels for 46 diseases.

**Main Outcomes and Measures:**

2019 NIH funding levels for 46 diseases.

**Results:**

The 46 diseases accounted for 62 392 713 of 94 399 784 disability-adjusted life-years (66.1%) in 2008 and 75 706 718 of 111 074 472 disability-adjusted life-years (68.2%) in 2019, representing more than 66% of all disability-adjusted life-years in both years. By dollar volume, Alzheimer and dementia increased the most, with approximately $1.8 billion more funding in 2019 than 2008 (from $530 million in 2008 to $2398 million in 2019, a 352% increase), whereas interpersonal violence had the greatest decrease, $95 million, in 2019 NIH funding (from $236 million in 2008 to $141 million in 2019, a 40% decrease). For the 46 diseases in this study, the variable with the greatest association with NIH funding in 2019 was the level of NIH funding in 2008, with a simple correlation of 0.88. Burden of disease and changes in burden of disease were not statistically significantly associated with NIH funding levels once the prior level of funding was included in the model. The models suggested that a 1% higher level of NIH funding in 2008 was associated with a 0.91% higher level of NIH funding in 2019.

**Conclusions and Relevance:**

In this study, NIH spending for most diseases seemed to be based primarily on the level of NIH spending more than 10 years earlier, despite changes in burden of disease. Congress and the NIH should examine the allocation process to ensure NIH investments are responsive to changes in the health of the population.

## Key Points

**Question:**

Are National Institutes of Health (NIH) funding levels associated with changes in burden of disease?

**Findings:**

This cohort study of 46 diseases found that NIH funding levels for a specific disease in 2019 were most highly associated with such levels in 2008. Burden of disease and changes in burden of disease were not statistically significantly associated with NIH funding levels once the prior level of funding was included in the model.

**Meaning:**

In this study, NIH spending appeared to be based primarily on the level of spending for that disease in prior years, despite changes in burden of disease.

## Teaser

This cohort study examines the factors associated with National Institutes of Health funding in 2019 for 46 diseases.

## Introduction

The National Institutes of Health (NIH) serves a dual mission, striving to support the next generation of fundamental scientific knowledge and the application of that knowledge to improve population health.^1^ Congress and the NIH allocate the budget for biomedical research to specific institutes and then often to specific centers and diseases, suggesting that the NIH is using burden of disease as a factor in making allocation decisions to diseases that are most likely to improve health for people living in the United States.

Other possible funding considerations include recent changes in the burden of disease, the potential for a scientific breakthrough, advocacy by disease-focused organizations, or simply an increase in funding based on the allocation for that disease in prior years. This allocation process is not immune to political influence; Congress determines how dollars are spent. The process can be considered part of a social contract: congressional influence on research spending in exchange for long-standing congressional support of the NIH budget. NIH funding has increased more slowly than growth in the gross domestic product in recent years.^2^ Hence, the rationale for NIH allocations for specific diseases is critical.

A 1999 study found a statistically significant correlation between the level of NIH funding and the burden of disease as measured by disability-adjusted life-years for 29 different diseases.^3^ The analysis also reported that certain diseases, most notably AIDS, diabetes, dementia, and breast cancer, received more NIH funding than would have been expected according to an allocation based solely on disability-adjusted life-years, whereas chronic obstructive pulmonary disease, perinatal conditions, and peptic ulcer received less than would be expected solely according to disability-adjusted life-years.

Since the 1999 article was published, studies have shown similar associations between burden of disease and NIH funding.^4,5^ However, during the past 20 years, the methods for measurement of burden of disease have improved, the burden of disease in the United States has changed, and there have been advances in basic science.

Therefore, an important question is whether recent changes in the burden of disease are reflected in the NIH disease-specific allocation process. In this article, we examine factors associated with 2019 NIH funding levels for specific diseases, including current and historical disease burden, cost of providing medical care, and prior levels of NIH funding.

## Methods

### Study Design

We compared NIH funding in 2008 and 2019 in both cross-sectional and longitudinal analyses for 46 diseases to identify which factors—2008 and 2019 burden of disease, 2016 health spending, and 2008 NIH funding levels for that disease—were most associated with NIH funding allocations for the 46 diseases in 2019. Our analysis involved nonhuman data and, per the Common Rule, was exempted from institutional review board review and the requirement for informed consent. The reporting of this study is in accordance with Strengthening the Reporting of Observational Studies in Epidemiology (STROBE) reporting guideline.

### Burden of Disease

We identified the 2008 and 2019 US burden of disease by using data from the Institute for Health Metrics and Evaluation database.^6^ Although multiple disease metrics were available, we chose percentage of the total number of US disability-adjusted life-years associated with each disease for 3 reasons. First, disability-adjusted life-years account for both morbidity and mortality and thereby provide a more holistic view of health burden than other burden of disease metrics, such as deaths. Second, a prior study showed that disability-adjusted life-years were the measure of burden of disease with the greatest association with NIH funding.^3^ Third, we used the percentage of total disability-adjusted life-years attributable to each disease to control for population change.

### NIH Funding

We then identified 2008 and 2019 NIH categoric funding. For both years, we used NIH’s Research, Condition, and Disease Categories funding data reported by the NIH Office of Budget.^7^ These data report annual funding for research grants, research and development contracts, and research conducted by NIH across 285 research areas and diseases. The 2008 NIH funding amounts were inflated to 2019 dollars with the Biomedical Research and Development Price Index.^8^ We matched Research, Condition, and Disease Categories data to 46 diseases with the US burden of disease data.

### Cost of Providing Health Care

We used estimated 2016 direct health care spending as a measure of resources allocated to certain diseases.^9^ These are the spending numbers that policy makers would have used to determine NIH funding levels at the time. The direct health care spending estimates are suggestive of total US health care spending and include amounts paid by insurers and patients. These values were inflated to 2019 dollars with the Consumer Price Index.^10^

### Statistical Analysis

We calculated total rates of change between 2008 and 2019 for both burden of disease and NIH funding for the 46 diseases. We used regression analysis to estimate the association between burden of disease (in 2008 or 2019), change in burden of disease (from 2008 to 2019), proportion of NIH funding spent on each disease in 2008, 2016 US health spending, and the 2019 NIH proportion of funding for the 46 diseases. We estimated 5 ordinary least squares regression models and presented the associations graphically. The first 4 regression models used bivariate analysis, with the explanatory variables being 2019 burden of disease, 2008 burden of disease, 2016 health spending, and 2008 NIH funding for models 1 to 4, respectively. Model 5 included all explanatory variables in the prior models. Given the nonnormal distribution of these variables, all were log transformed for the regression analysis. To test for statistical significance of coefficients, we used 2-sided hypothesis tests with a priori α = .05. All statistical analysis was conducted with Stata version 16.1 (StataCorp). We performed the same analysis for the 29 diseases used in the 1999 study^3^ to obtain a longer time perspective, albeit with fewer diseases.

## Results

### Burden of Disease in Disability-Adjusted Life-Years

Table 1 shows the percentage of total US disability-adjusted life-years for the 46 diseases in both 2008 and 2019. 62 392 713 of 94 399 784 disability-adjusted life-years (66.1%) in 2008 and 75 706 718 of 111 074 472 disability-adjusted life-years (68.2%) in 2019, representing more than 66% of all disability-adjusted life-years in both years. There were substantial changes in burden of disease between 2008 and 2019; for example, the proportion of disability-adjusted life-years attributable to drug use disorders increased 58% (from 3.5% to 5.5%), whereas that attributable to HIV/AIDS decreased 40% (from 0.6% to 0.4%).

**Table 1.** US Burden of Disease, 2008 and 2019

Disease category	Burden, DALYs, No. (%)		Change in proportion of total DALYs, %
	2008 (N = 94 399 784)	2019 (N = 111 074 472)	
Total	62 392 713 (66.1)	75 706 718 (68.2)	3.1
Cardiovascular			
Total	10 888 855 (11.6)	12 281 658 (11.1)	–4.1
Ischemic heart disease	8 189 345 (8.7)	8 948 089 (8.1)	–7.1
Other cardiovascular diseases	1 857 071 (2.0)	2 257 802 (2.0)	3.3
Hypertension	842 439 (0.9)	1 075 767 (1.0)	8.5
Other			
Total	9 728 499 (10.3)	9 787 097 (8.8)	–14.5
Injuries	3 042 552 (3.2)	2 954 759 (2.7)	–17.5
Perinatal disorders^a^	1 897 649 (2.0)	1 589 864 (1.4)	–28.8
Self-harm	1 779 977 (1.9)	1 903 093 (1.7)	–9.1
Interpersonal violence	1 267 997 (1.3)	1 197 087 (1.1)	–19.8
Oral disorders	846 546 (0.9)	198 547 (1.1)	20.3
Urologic diseases	362 297 (0.4)	453 761 (0.4)	6.4
Psoriasis	326 408 (0.3)	353 965 (0.3)	–8.1
Sudden infant death syndrome	205 073 (0.2)	136 021 (0.1)	–43.6
Cancer			
Total	9 594 290 (10.2)	3 057 849 (10.0)	–2.1
Lung cancer	3 807 647 (4.0)	4186491 (3.8)	–6.5
Colon and rectum cancer	1 459 687 (1.6)	1760640 (1.6)	2.5
Breast cancer	1 292 302 (1.4)	1 403 392 (1.3)	–7.7
Prostate cancer	714 001 (0.8)	926 635 (0.8)	10.3
Leukemia	604 098 (0.6)	668 595 (0.6)	–5.9
Non-Hodgkin lymphoma	525 735 (0.6)	607 660 (0.5)	–1.8
Ovarian cancer	394 723 (0.4)	426 504 (0.4)	–8.2
Liver cancer	371 578 (0.4)	551 263 (0.5)	26.1
Cervical cancer	198 102 (0.2)	247 830 (0.2)	–3.6
Uterine cancer	175 262 (0.2)	249 557 (0.2)	21.0
Hodgkin lymphoma	51 155 (0.1)	29 282 (<0.1)	–18.1
Mental health			
Total	7 586 375 (8.0)	10 606 093 (9.5)	18.9
Drug use disorders	3 291 046 (3.5)	6 121 628 (5.5)	58.2
Depressive disorders	2 620 507 (2.8)	2 652 532 (2.4)	–14.0
Alcohol use disorders	1 144 800 (1.2)	1 275 578 (1.1)	–5.3
Eating disorders	273 056 (0.3)	261 295 (0.2)	–18.7
Autism	256 966 (0.3)	295 060 (0.3)	–2.4
Neurologic			
Total	6 643 060 (7.0)	7 775 109 (7.0)	–0.5
Stroke	3 279 739 (3.5)	3 826 274 (3.5)	–0.8
Alzheimer and dementias	1 652 481 (1.8)	2 026 882 (1.8)	4.3
Schizophrenia	981 296 (1.0)	993 335 (0.9)	–13.9
Parkinson disease	369 196 (0.4)	492 368 (0.4)	13.4
Multiple sclerosis	186 529 (0.2)	211 385 (0.2)	–3.7
Motor neuron disease	173 819 (0.2)	224 865 (0.2)	10.0
Respiratory			
Total	6 195 089 (6.6)	7 664 787 (6.9)	4.2
COPD	4 097 818 (4.4)	5 021 538 (4.5)	4.2
Pneumonia	1 017 898 (1.1)	1 228 694 (1.1)	–2.6
Asthma	1 079 373 (1.1)	1 414 555 (1.3)	11.4
GI			
Total	4 020 808 (4.3)	5 023 554 (4.5)	6.2
Chronic kidney disease	1 726 275 (1.8)	2 287 706 (2.1)	12.7
Cirrhosis	1 532 849 (1.6)	1 825 800 (1.7)	1.2
Digestive disorders	578 159 (0.6)	694 759 (0.6)	2.1
Inflammatory bowel disease	183 525 (0.2)	215 289 (0.2)	–0.3
Endocrine			
Total (diabetes)	3 492 035 (3.7)	4 461 171 (4.0)	8.5
Infection			
Total	655 999 (0.8)	487 949 (0.5)	–32.2
HIV/AIDS	585 527 (0.6)	415 326 (0.4)	–39.8
STIs, excluding HIV	45 413 (0.05)	47 592 (0.04)	0.0
Tuberculosis	25 059 (0.03)	25 031 (0.02)	–11.0
Sensory			
Total (otitis media)	47 834 (0.05)	50 140 (0.04)	–10.9

Abbreviations: COPD, chronic obstructive pulmonary disease; DALY, disability-adjusted life-year; GI, gastrointestinal; STI, sexually transmitted infection.

^a^ Includes neonatal disorders.

### NIH Funding Levels

Table 2 shows the proportion and level of NIH funding for the 46 diseases in 2008 and 2019. The inflation-adjusted overall funding increase for all 46 diseases during the 11 years was 10%. However, certain diseases experienced higher NIH funding growth rates than others. Adjusted for inflation, Alzheimer and dementia, leukemia, tuberculosis, and self-harm had NIH funding increases greater than 100%, whereas sudden infant death syndrome, interpersonal violence, multiple sclerosis, Hodgkin lymphoma, and otitis media had funding decreases of at least 40%. By dollar volume, Alzheimer and dementia increased the most, with approximately $1.8 billion more funding in 2019 than 2008 (from $530 million in 2008 to $2398 million in 2019, a 352% increase), whereas interpersonal violence had the greatest decrease, $95 million, in 2019 NIH funding (from $236 million in 2008 to $141 million in 2019, a 40% decrease).

**Table 2.** NIH Funding for 2008 and 2019 and Proportion of Total NIH Funding

Disease category	NIH spending, No. (%), $ (in millions)		Change %
	2008 (N = 37 735)^a^	2019 (N = 39 420)	
Total	21 512 (57.0)	23 680 (60.1)	10
Infection			
Total	4438 (11.8)	4075 (10.3)	–8
HIV/AIDS	3770 (10.0)	3037 (7.7)	–19
STIs, excluding HIV	315 (0.8)	354 (0.9)	12
Tuberculosis	183 (0.5)	488 (1.2)	167
Malaria	170 (0.5)	196 (0.5)	15
GI			
Total	2924 (7.7)	3336 (8.5)	14
Digestive diseases	1836 (4.9)	2173 (5.5)	18
Chronic kidney disease	673 (1.8)	649 (1.6)	–4
Cirrhosis	310 (0.8)	351 (0.9)	13
Inflammatory bowel disease	104 (0.3)	163 (0.4)	56
Other			
Total	2792 (7.4)	3125 (7.9)	12
Urologic diseases	687 (1.8)	546 (1.4)	–21
Injuries	597 (1.6)	897 (2.3)	50
Oral disorders	596 (1.6)	613 (1.6)	3
Perinatal disorders^b^	578 (1.5)	784 (1.9)	36
Interpersonal violence	236 (0.6)	141 (0.4)	–40
Self-harm	50 (0.1)	117 (0.3)	133
Sudden infant death syndrome	37 (0.1)	11 (<0.1)	–71
Psoriasis	10 (<0.1)	16 (<0.1)	55
Cardiovascular			
Total	2610 (6.9)	2394 (6.1)	–8
Ischemic heart disease	1567 (4.2)	1443 (3.7)	–8
Other cardiovascular diseases	704 (1.9)	685 (1.7)	–3
Hypertension	339 (0.9)	266 (0.7)	–21
Mental health			
Total	2557 (6.8)	3056 (7.8)	20
Drug use disorders	1296 (3.4)	1621 (4.1)	25
Alcohol use disorders	582 (1.5)	556 (1.4)	–4
Depressive disorders	518 (1.4)	578 (1.5)	12
Autism	152 (0.4)	290 (0.7)	91
Eating disorders	9 (<0.1)	11 (<0.1)	22
Cancer			
Total	2545 (6.7)	2560 (6.5)	1
Breast cancer	935 (2.5)	709 (1.8)	–24
Prostate cancer	373 (1.0)	263 (0.7)	–30
Colon and rectum cancer	353 (0.9)	294 (0.7)	–17
Non-Hodgkin lymphoma	248 (0.7)	248 (0.6)	0
Lung cancer	218 (0.6)	419 (1.1)	93
Ovarian cancer	124 (0.3)	168 (0.4)	36
Liver cancer	115 (0.3)	127 (0.3)	11
Cervical cancer	89 (0.2)	106 (0.3)	19
Leukemia	50 (0.1)	178 (0.5)	255
Hodgkin lymphoma	21 (0.1)	12 (<0.1)	–42
Uterine cancer	21 (0.1)	36 (0.1)	75
Neurologic			
Total	1701 (4.5)	3451 (8.8)	103
Alzheimer and dementias	530 (1.4)	2398 (6.1)	352
Stroke	381 (1.0)	350 (0.9)	–8
Schizophrenia	321 (0.8)	263 (0.7)	–18
Multiple sclerosis	218 (0.6)	111 (0.3)	–49
Parkinson disease	196 (0.5)	224 (0.6)	14
Motor neuron disease	55 (0.1)	105 (0.3)	90
Endocrine			
Total (diabetes)	1390 (3.7)	1099 (2.8)	–21
Respiratory			
Total	533 (1.4)	571 (1.4)	7
Asthma	317 (0.8)	313 (0.8)	–1
Pneumonia	120 (0.3)	146 (0.4)	22
COPD	97 (0.3)	112 (0.3)	16
Sensory			
Total (otitis media)	23 (0.1)	13 (<0.1)	–44

Abbreviations: COPD, chronic obstructive pulmonary disease; GI, gastrointestinal; STI, sexually transmitted infection.

^a^ Adjusted to 2019 dollars.

^b^ Includes neonatal disorders.

### Association of Disability-Adjusted Life-Years With Change to NIH Funding Levels

**Figure 1.**
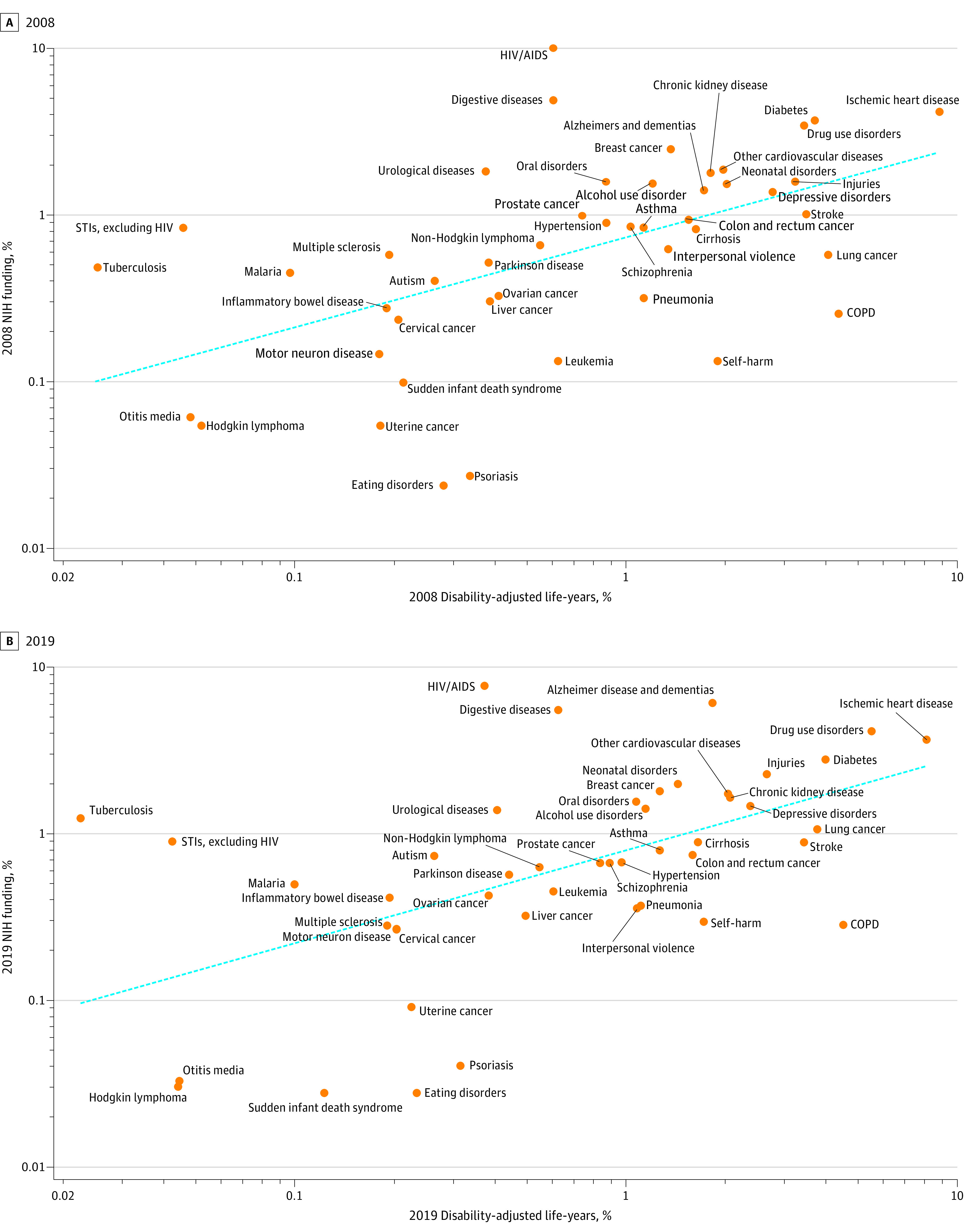
National Institutes of Health (NIH) Funding and Burden of Disease Funding and disease burden in 2008 (A) and 2019 (B). The dashed lines indicate the fitted association between burden of disease and NIH spending. COPD indicates chronic obstructive pulmonary disease and STI, sexually transmitted infection.

There was a positive association between disability-adjusted life-years and NIH funding in 2008 and 2019 (Figure 1A and B). The fitted line had a coefficient of 0.54 and 0.55 in 2008 and 2019, respectively. The simple correlation between disability-adjusted life-years and NIH funding was 0.30 in 2008 and 0.34 in 2019. In both years, 3 diseases received substantially more NIH funding than could be explained according to disability-adjusted life-years alone (HIV/AIDS, digestive diseases, and urologic diseases) and 3 diseases received relatively less funding than would be expected according to disability-adjusted life-years (eating disorders, uterine cancer, and psoriasis). Most of the other diseases received NIH allocations close to the expected value according to the number of disability-adjusted life-years in both years. In 2009, the American Recovery and Reinvestment Act provided additional money to the NIH outside of the normal allocation process. Funding for the 46 diseases in this allocation process was similar to the allocation using the normal allocation process (simple correlation, 0.35) (eFigure 3 in the Supplement).

**Figure 2.**
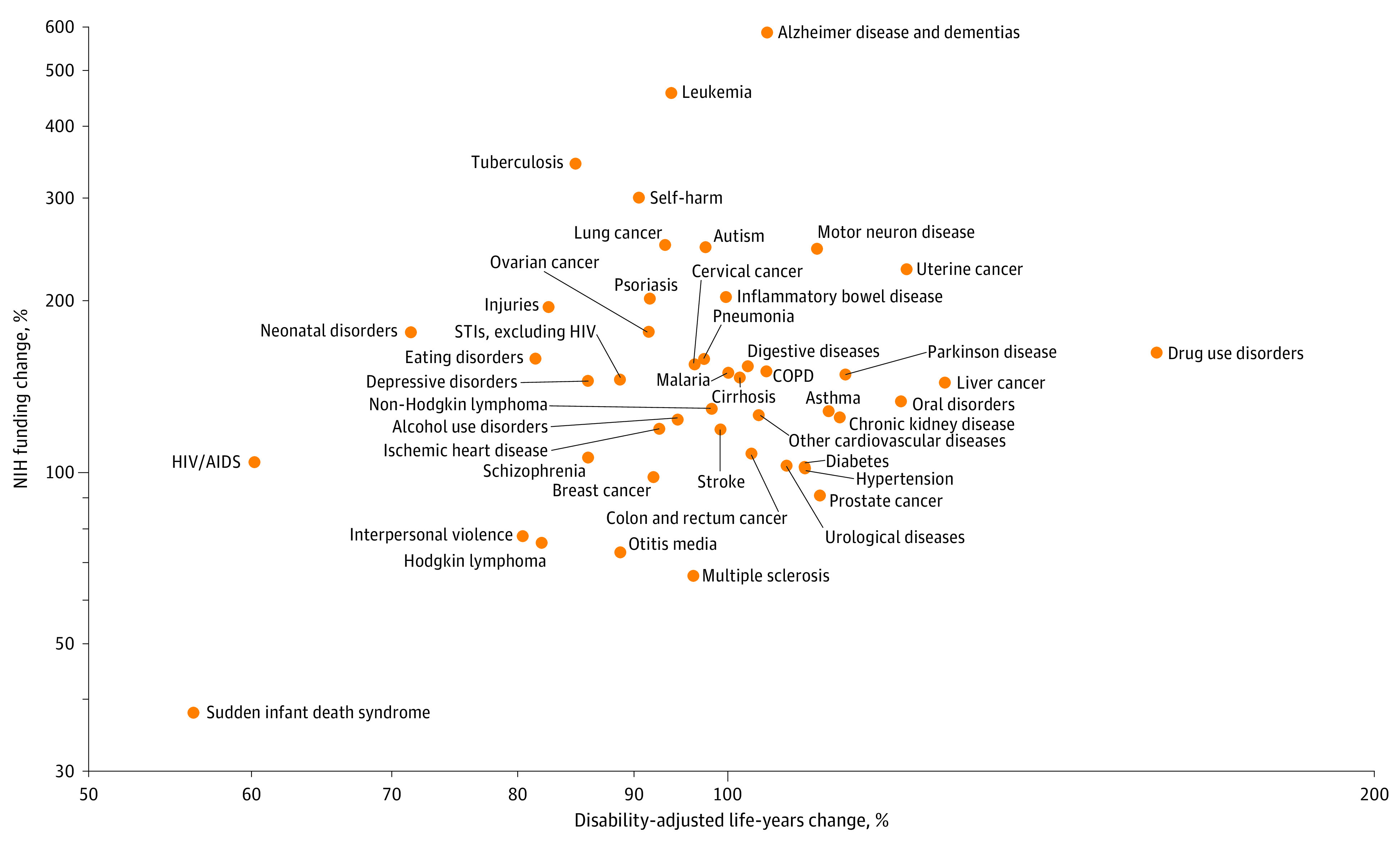
Changes in Burden of Disease and National Institutes of Health (NIH) Funding, 2008-2019 COPD indicates chronic obstructive pulmonary disease and STI, sexually transmitted infection.

We examined the changes in burden of disease between 2008 and 2019 and the changes in NIH funding during the same period (Figure 2). Diseases with a larger increase in disability-adjusted life-years during this period did not necessarily receive a larger increase in NIH funding (simple correlation, 0.08).

### Association of Disability-Adjusted Life-Years With 2019 NIH Funding Levels for 46 Conditions

When only 2019 burden of disease was used to explain NIH funding for each of the 46 diseases, 29% of the overall variance in NIH funding was explained, and increases in 2019 disability-adjusted life-years were associated with increases in NIH funding (Table 3, model 1). When only 2008 burden of disease was used to explain NIH funding, the amount of overall variance in 2019 funding decreases was similar, at 28% (Table 3, model 2). When only estimated 2016 health spending for each disease was used, the amount of overall variance in NIH funding explained was 25% (model 3).

**Table 3.** Factors Associated With 2019 NIH Funding for 46 Diseases

Variable	Coefficient (SE)^a^				
	Model 1	Model 2	Model 3	Model 4	Model 5
2019 DALY	0.546 (0.128)^b^	NA	NA	NA	0.554 (0.447)
2008 DALY	NA	0.552 (0.133)^b^	NA	NA	–0.539 (0.451)
2016 Health spending	NA	NA	0.334 (0.0865)^b^	NA	0.0465 (0.0444)
2008 NIH funding	NA	NA	NA	0.958 (0.0538)^b^	0.910 (0.0639)^b^
Observations	46	46	46	46	46
*R*^2^	0.292	0.281	0.253	0.878	0.891

Abbreviations: DALY, disability-adjusted life-year; NIH, National Institutes of Health; NA, not applicable.

^a^ Coefficients should be interpreted as elasticities. For example, in model 1, the coefficient for 2019 DALYs should be interpreted as a 1% increase in 2019 DALYs yields a 0.546% increase in 2019 NIH spending. Constants are excluded.

^b^ *P* < .01.

### Association of 2008 NIH Funding With 2019 NIH Funding Levels for 46 Conditions

Using 2008 NIH funding, the overall explained variance was almost 3 times higher than that of other models (Table 3, model 4). Including this variable in the model with the other explanatory variables explained more than 89% of the variance, and the other variables became statistically insignificant (Table 3, model 5). The coefficient for 2008 NIH funding of 0.91 suggests that a 0.9% increase in relative 2008 NIH funding was associated with a 1% increase in relative 2019 NIH funding. The simple correlation between 2008 and 2019 NIH funding for the 46 diseases was 0.88.

As a sensitivity analysis, we compared the ability of 1996 NIH funding and 2019 disability-adjusted life-years to estimate 2019 NIH funding for the same 29 diseases in the 1999 study.^3^ The results were similar to the 2008 to 2019 data (eFigure 1, eFigure 2, and eTables 1-4 in the Supplement).

## Discussion

The strongest estimator of NIH funding in 2019 for 46 diseases was the level of NIH funding for that condition 11 years earlier. When the period was extended to 23 years, a similar result was found for 29 diseases. The burden of disease (as measured by disability-adjusted life-years) was associated with the level of funding, but once prior levels of NIH funding were included in the model, the burden of disease was not statistically significant. Changes in the burden of disease did not track with the level of NIH funding for these 46 diseases.

Researchers have suggested that other factors could influence the level of NIH funding for specific diseases, including percentage of attributable deaths, global disease burden, public health needs, scientific opportunities, quality of research proposals, and maintenance of staffing.^5,11,12^ Moreover, basic science research can yield fundamental discoveries that affect human disease unpredictably, and insights developed from research into 1 disease might end up having a greater influence on other conditions.

That prior funding had the strongest association with current disease-specific funding requires a brief summary of the allocation process. The current allocation process begins with the NIH director working with the directors of the 27 institutes and centers to develop a preliminary budget. In consultation with the US Department of Health and Human Services and Office of Management and Budget, the NIH prepares the president's budget and congressional justification. Congress uses this information to appropriate funding for specific initiatives and centers within the NIH. Therefore, the question is why has the allocation of NIH funding across conditions remained relatively static and why does it not appear to be responsive to changing burden of disease? For the institutes and centers, we compared the levels of funding in 2008 and 2019 and found that they were highly correlated (eFigure 3 in the Supplement).

A commonly cited factor for NIH allocation decisions is scientific opportunity.^11,13^ We agree that centers and institutes are typically looking for the best and most innovative research; however, an important question is whether research on the same diseases remains on the forefront of discovery for many years. It is difficult to accept, given the constancy of funding across diseases, that the relative likelihood of scientific breakthroughs varies in the same way across diseases now as it did 11 or 21 years earlier.

Disease-specific advocacy also plays an important role in NIH funding.^14^ Although advocates’ success in garnering congressional support for research can lead to higher overall NIH budgets, most advocacy groups focus on specific diseases. Some of the extra funding that certain diseases obtain could be the result of these efforts.

There has been great stability in the funding of diseases during the last 11 and 21 years. A former NIH director has written a book suggesting some of the difficulties the leadership of the NIH and Congress have in making allocation decisions.^12,15^ There are many reasons for Congress and the NIH to continue spending at levels similar to those of past years. Possibly the most important is that there is an infrastructure of people at the NIH and researchers in academic medical centers who have invested substantial human capital in certain diseases and may advocate maintaining funding.

A number of organizations have suggested allocation revisions to NIH funding. A 1998 Institute of Medicine report emphasized 5 criteria: public health needs, quality of research supported, scientific opportunity, portfolio diversification, and adequate infrastructure support.^11^ Although it appears that the NIH is committed to portfolio diversification, the small change in relative funding priorities raises concerns that other criteria are being ignored.

### Limitations

There are limitations to this study. First, research is hard to parse into specific categories of disease, particularly basic science, infrastructure support, and research or interventions that bridge disciplines (eg, a study of breast cancer in persons with diabetes). The 46 diseases account for 60% of NIH funding and represented 85% of NIH research grant funding in 2019. The remaining funding is for administration, other diseases, and for activities that are not specific to a disease. Another limitation is the reliance on disability-adjusted life-years to measure burden of disease. We cannot assess how prior funding for certain disease categories has changed the burden of disease. Third, the regression results suggest that other factors, such as scientific opportunities and lobbying by advocacy organizations, can play a role in determining how much funding a specific disease receives.

## Conclusions

In this study, the distribution of NIH funding for 46 specific diseases in 2019 was remarkably similar to that observed in 2008 (and for 29 diseases in 1996), despite changes in burden of disease. There was an association between burden of disease and NIH funding; however, the factor most strongly associated with NIH funding in 2019 was the level of such funding in 2008 (also 1996), suggesting that changing public health needs have a limited role in guiding NIH funding decisions. Congress and the NIH should examine the factors and processes they use to allocate funds to specific diseases to ensure that taxpayer dollars are being used to achieve the current needs for human health.

## Notes

Supplement. **eTable 1.** US Burden of Disease 1996 and 2019 for 29 Diseases **eTable 2.** NIH Funding 1996 and 2019 **eFigure 1.** Burden of Disease and NIH Funding 1996 and 2019 **eFigure 2.** Changes in Burden of Disease and NIH Funding (1996-2019) **eTable 3.** Explaining 2019 NIH Funding Using Change in Disease Burden and 2008 NIH Funding **eTable 4.** Explaining 2019 NIH Funding for 29 Diseases **eFigure 3.** Correlation Between 2008 and 2019 Institutes and Centers Funding
