# Supplementary material for: Allocation of National Institutes of Health Funding by Disease Category in 2008 and 2019
Source: JAMA Netw Open. 2021 Jan 27;4(1):e2034890. doi: 10.1001/jamanetworkopen.2020.34890 (PMC7841468; doi:10.1001/jamanetworkopen.2020.34890)
Supplement: Supplement. — eTable 1. US Burden of Disease 1996 and 2019 for 29 Diseases eTable 2. NIH Funding 1996 and 2019 eFigure 1. Burden of Disease and NIH Funding 1996 and 2019 eFigure 2. Changes in Burden of Disease and NIH Funding (1996-2019) eTable 3. Explaining 2019 NIH Funding Using Change in Disease Burden and 2008 NIH Funding eTable 4. Explaining 2019 NIH Funding for 29 Diseases eFigure 3. Correlation Between 2008 and 2019 Institutes and Centers Funding [file jamanetwopen-e2034890-s001.pdf]

Supplemental Online Content

Ballreich JM, Gross CP, Powe NR, Anderson GF. Allocation of National Institutes of Health funding by disease category in 2008 and 2019. *JAMA Netw Open*. 4(1):e2034890. doi:10.1001/jamanetworkopen.2020.34890

eTable 1. US Burden of Disease 1996 and 2019 for 29 Diseases

eTable 2. NIH Funding 1996 and 2019

eFigure 1. Burden of Disease and NIH Funding 1996 and 2019

eFigure 2. Changes in Burden of Disease and NIH Funding (1996-2019)

eTable 3. Explaining 2019 NIH Funding Using Change in Disease Burden and 2008 NIH Funding

eTable 4. Explaining 2019 NIH Funding for 29 Diseases

eFigure 3. Correlation Between 2008 and 2019 Institutes and Centers Funding

This supplemental material has been provided by the authors to give readers additional information about their work.

© 2021 Ballreich JM et al. *JAMA Network Open.*

eTable 1. U.S. Burden of Disease 1996 and 2019 for 29 Diseases

Disease Category	1996 Burden (Proportion of total US DALYs)	2019 Burden (Proportion of total US DALYs)	Change
Total	51.1%	45.3%	-11.3%
Cardiovascular	11.5%	8.1%	-29.5%
Ischemic Heart Disease	11.5%	8.1%	-29.5%
Cancer	9.2%	8.3%	-10.3%
Lung Cancer	4.4%	3.8%	-13.4%
Colon And Rectum Cancer	1.6%	1.6%	-2.9%
Breast Cancer	1.6%	1.3%	-19.4%
Prostate Cancer	0.9%	0.8%	-3.7%
Ovarian Cancer	0.4%	0.4%	-7.5%
Cervical Cancer	0.2%	0.2%	-10.1%
Uterine Cancer	0.2%	0.2%	43.7%
Neurological	7.2%	7.1%	-0.7%
Stroke	3.8%	3.5%	-8.4%
Alzheimer’s And Dementias	1.6%	1.8%	14.1%
Schizophrenia	1.0%	0.9%	-9.2%
Epilepsy	0.3%	0.3%	-10.7%
Parkinson's Disease	0.3%	0.4%	47.4%
Multiple Sclerosis	0.2%	0.2%	15.2%
Other	7.1%	5.2%	-27.1%
Injuries	3.9%	2.7%	-32.4%
Neonatal Disorders	2.1%	1.4%	-31.6%
Oral Disorders	1.1%	1.1%	1.1%
Respiratory	6.2%	6.9%	12.3%
COPD	3.7%	4.5%	23.1%
Pneumonia	1.5%	1.1%	-24.7%
Asthma	1.0%	1.3%	27.2%
Mental Health	3.6%	3.5%	-2.6%
Depressive Disorders	2.5%	2.4%	-4.8%
Alcohol Use Disorders	1.1%	1.1%	2.5%
Endocrine	2.6%	4.0%	52.9%
Diabetes	2.6%	4.0%	52.9%
Infectious Diseases	2.2%	0.4%	-79.6%
HIV/AIDS	2.0%	0.4%	-81.7%
STIs ex HIV	0.1%	0.0%	-21.5%
Tuberculosis	0.1%	0.0%	-56.6%
GI	1.5%	1.7%	13.8%
Cirrhosis	1.4%	1.7%	21.5%
Peptic Ulcer	0.2%	0.1%	-45.4%
Sensory	0.1%	0.0%	-18.2%
Otitis Media	0.1%	0.0%	-18.2%

© 2021 Ballreich JM et al. *JAMA Network Open.*

eTable 2. NIH Funding 1996 and 2019

Disease Category	1996 NIH Spending ($ millions)*	2019 NIH Spending ($ millions)	Change
Total	$9,750	$15,616	67%
Infectious Diseases	$3,124	$3,879	24%
HIV/AIDs	$2,794	$3,037	9%
STIIs Ex HIV	$203	$354	74%
Tuberculosis	$127	$488	284%
Cancer	$1,632	$1,995	22%
Breast Cancer	$756	$709	-6%
Lung Cancer	$253	$419	66%
Colon and Rectum Cancer	$209	$294	41%
Prostate Cancer	$183	$263	43%
Cervical Cancer	$119	$106	-11%
Ovarian Cancer	$83	$168	101%
Uterine Cancer	$28	$36	30%
Neurological	$1,487	$3,534	138%
Alzheimer's And Dementias	$603	$2,398	298%
Stroke	$238	$350	47%
Schizophrenia	$221	$263	19%
Multiple Sclerosis	$164	$111	-32%
Parkinson's Disease	$153	$224	47%
Epilepsy	$109	$188	72%
Other	$816	$1,589	95%
Injuries	$393	$897	128%
Oral Disorders	$370	$613	65%
Neonatal Disorders	$52	$79	51%
Mental Health	$793	$1,134	43%
Alcohol Use Disorders	$508	$556	9%
Depressive Disorders	$285	$578	103%
Endocrine	$592	$1,099	86%
Diabetes	$592	$1,099	86%
Cardiovascular	$533	$1,443	171%
Ischemic Heart Disease	$533	$1,443	171%
Respiratory	$408	$571	40%
Asthma	$162	$313	94%
COPD	$124	$112	-9%
Pneumonia	$123	$146	19%
GI	$348	$359	3%
Cirrhosis	$336	$351	4%
Peptic Ulcer	$12	$8	-33%
Sensory	$18	$13	-28%
Ottis Media	$18	$13	-28%

*Adjusted to 2019 dollars

© 2021 Ballreich JM et al. *JAMA Network Open.*

eFigure 1. Burden of Disease and NIH Funding 1996 and 2019

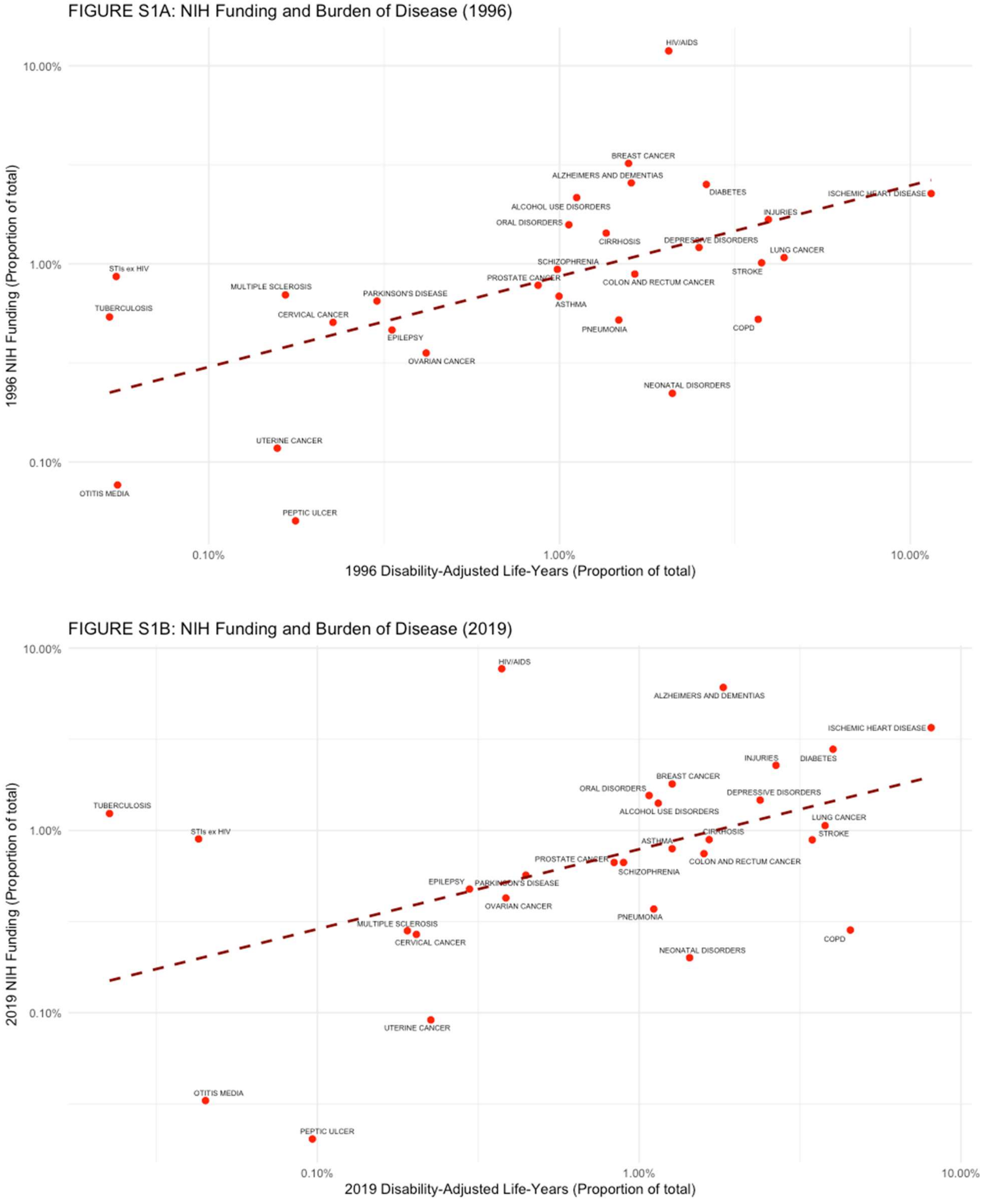

© 2021 Ballreich JM et al. *JAMA Network Open.*

eFigure 2. Changes in Burden of Disease and NIH Funding (1996 - 2019)

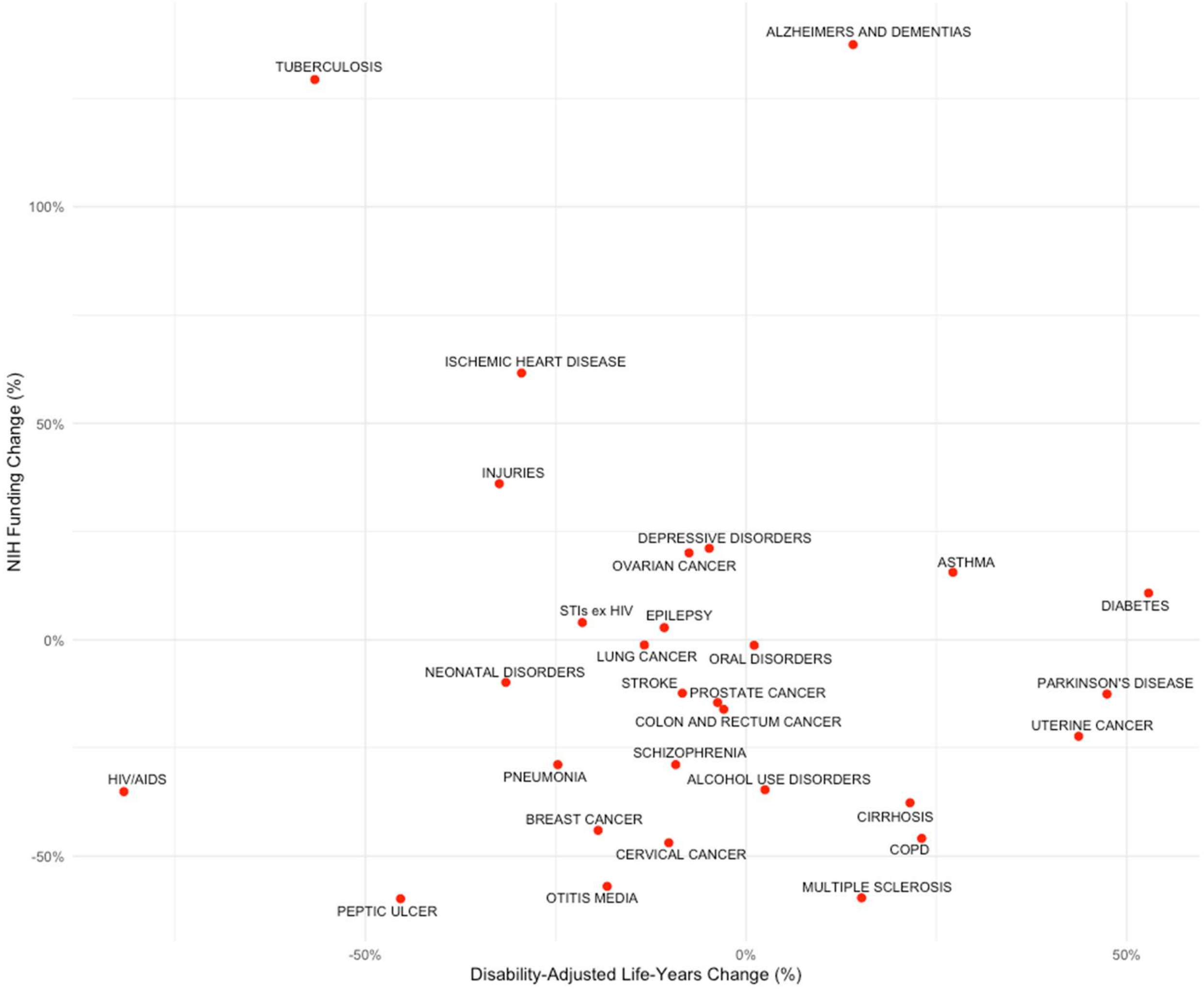

Linear OLS suggests R-squared<.01 and coefficient of DALY to be not statistically significant (p~0.81)

© 2021 Ballreich JM et al. *JAMA Network Open.*

eTable 3. Explaining 2019 NIH Funding using change in Disease Burden and 2008 NIH Funding

	(1)
VARIABLES	Model1
2008 NIH Funding	0.946***
	(0.0537)
Change in DALY	0.662
	(0.439)
Observations	46
R-squared	0.884

Standard errors in parentheses
*** p<0.01, ** p<0.05, * p<0.1

© 2021 Ballreich JM et al. *JAMA Network Open.*

eTable 4. Explaining 2019 NIH Funding for 29 Diseases

	(1)	(2)	(3)	(4)	(5)
VARIABLES	Model1	Model2	Model3	Model4	Model5
2019 DALY	0.463***				-0.0691
	(0.147)				(0.309)
2008 DALY		0.563***			0.281
		(0.144)			(0.332)
2016 Health Spend			0.318**		-0.133
			(0.153)		(0.104)
2008 NIH Funding				1.046***	0.973***
				(0.106)	(0.137)
Observations	29	29	29	29	29
R-squared	0.268	0.362	0.139	0.782	0.810

Standard errors in parentheses
*** p<0.01, ** p<0.05, * p<0.1

Coefficients should be interpreted as elasticities. For example, in Model 1, the coefficient for 2019 DALYs should be interpreted as a 1% increase in 2019 DALYs yields a 0.463% in 2019 NIH Spending. Constants are excluded.

© 2021 Ballreich JM et al. *JAMA Network Open.*

eFigure 3. Correlation between 2008 and 2019 Institutes and Centers Funding

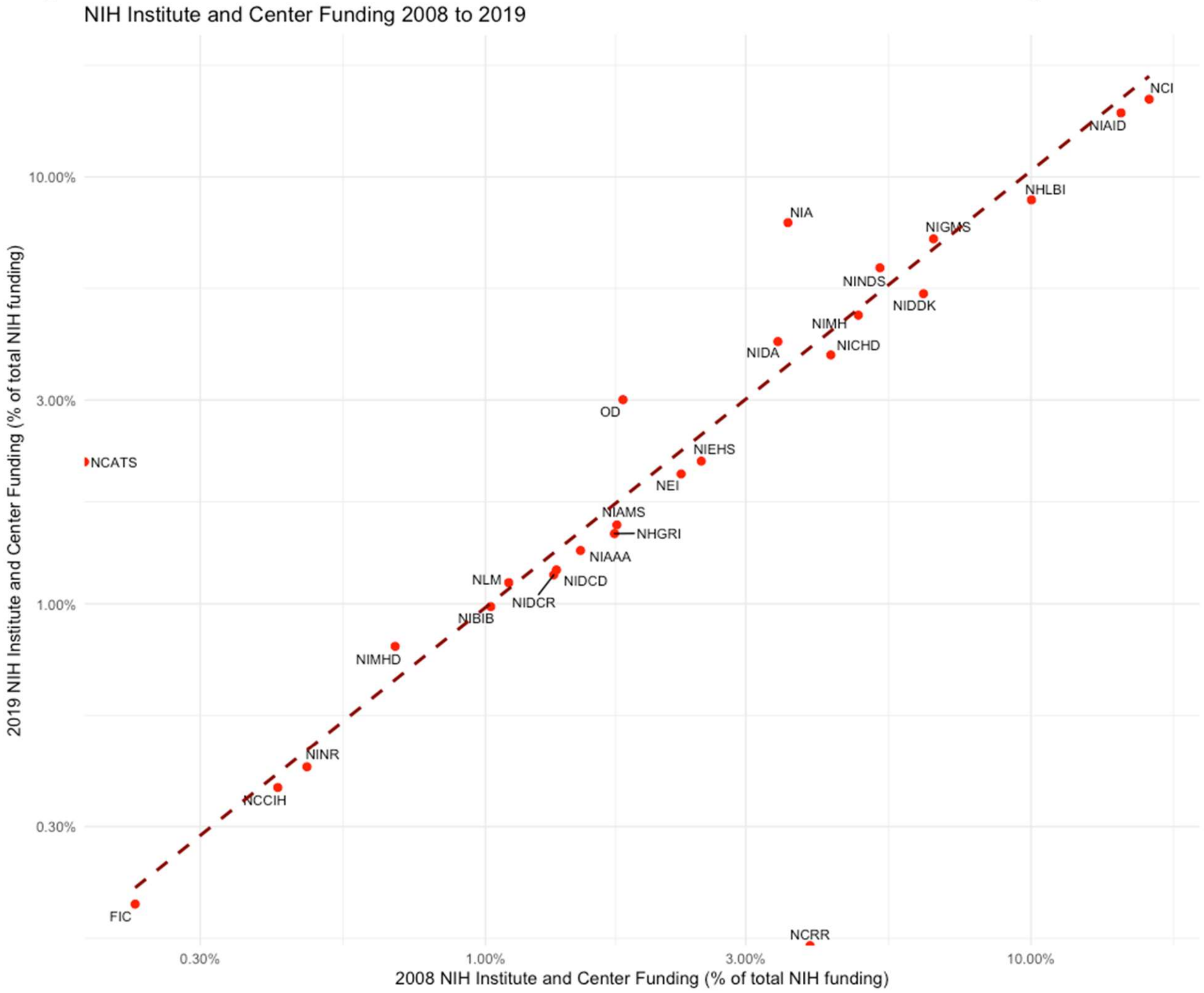

© 2021 Ballreich JM et al. *JAMA Network Open.*
